# Research on Carbon Emission Efficiency Space Relations and Network Structure of the Yellow River Basin City Cluster

**DOI:** 10.3390/ijerph191912235

**Published:** 2022-09-27

**Authors:** Haihong Song, Liyuan Gu, Yifan Li, Xin Zhang, Yuan Song

**Affiliations:** Urban and Rural Planning, School of Landscape Architecture, Northeast Forestry University, Harbin 150040, China

**Keywords:** Yellow River Basin, city cluster, carbon emission efficiency, spatial relationships, network structure

## Abstract

The Yellow River Basin serves as China’s primary ecological barrier and economic belt. The achievement of the Yellow River Basin’s “double carbon” objective is crucial to China’s green and low-carbon development. This study examines the spatial link and network structure of city cluster carbon emission efficiency in the Yellow River Basin, as well as the complexity of the network structure. It focuses not only on the density and centrality of the carbon emission efficiency network from the standpoint of city clusters, but also on the excellent cities and concentration of the city cluster ‘s internal carbon emission efficiency network. The results show that: (1) The carbon emission efficiency of the Yellow River Basin has been dramatically improved, and the gap between city clusters is narrowing. However, gradient differentiation characteristics between city clusters show the Matthew effect. (2) The distribution of carbon emission efficiency in the Yellow River Basin is unbalanced, roughly showing a decreasing trend from east to west. Lower-level efficiency cities have played a significant role in the evolution of carbon emissions efficiency space. (3) The strength of the carbon emission efficiency network structure in the Yellow River Basin gradually transitions from weakly correlated dominant to weakly and averagely correlated dominant. Among them, the Shandong Peninsula city cluster has the most significant number of connected nodes in the carbon emission efficiency network. In contrast, the emission efficiency network density of the seven city clusters shows different changing trends. Finally, this study suggests recommendations to improve carbon emission efficiency by adopting differentiated governance measures from the perspective of local adaptation and using positive spatial spillover effects.

## 1. Introduction 

In the international political and economic sphere, the issue of climate change is currently the focus of global research. To address this challenge, the United Nations has continued to promote global cooperation and clarify the responsibilities of developed and developing countries in reducing carbon dioxide emissions. As a responsible developing nation, China committed at the 75th session of the United Nations General Assembly to peak its carbon dioxide emissions by 2030 and move toward carbon neutrality by 2060. Nonetheless, because China is a huge nation with regional differences in economic structure, resource endowment, and institutional environment, it faces a severe carbon emission reduction issue [1]. Among them these differences, nine provinces (regions) in the Yellow River Basin accounted for 35.1% of the country’s total fossil energy consumption and 40.5% of the country’s carbon emissions in 2019 [2]. Therefore, the Yellow River Basin is a special region that must reduce carbon emissions immediately. 

Regional disparities in carbon emissions are evident in the Yellow River Basin, and the degree of economic growth has always had the greatest impact on the spatial heterogeneity of carbon emissions [3]. However, to thoroughly examine the geographic diversity of carbon emissions, it is necessary to consider both the degree of economic growth and the prospects for carbon emission reduction; therefore, the carbon emission efficiency with both characteristics is chosen as a reasonable evaluation metric [4]. The dual features refer to the intensity of carbon emissions constrained by economic conditions and the economic performance constrained by carbon emissions [5]. In order to describe the geographical pattern of carbon emission and the growth space of emission reduction in the Yellow River Basin, carbon emission efficiency is crucial. However, the current description of the spatial pattern of carbon emission efficiency is restricted to geographical space. It is only a qualitative analysis, which does not relieve all limitations of field space theory, and it is not easy to describe the network structure and correlation characteristics [6]. However, social network analysis based on relational data can reveal the association structure and attribute features of network members, thereby compensating for the shortcomings of conventional measurement research [7]. First, the majority of research objectives include total carbon emissions, because invisible carbon emissions obscure the flow of variables. Nevertheless, carbon emission efficiency can take into consideration both the technical efficiency of carbon emission production and the energy consumption efficiency of production activities, i.e., a thorough synthesis of the input and output elements of the production process [8]. Lastly, there is an absence of a comprehensive investigation of the network of carbon emission sources. The majority of past research has focused on agglomeration characteristics and spatial organization while ignoring the structural characteristics of people within a population.

Current research on carbon emission efficiency focuses primarily on the calculation of carbon emission efficiency, the variables that influence carbon emission efficiency, and the temporal and spatial development of carbon emission efficiency [9,10,11]. There are primarily two methods for calculating carbon emission efficiency: single components and entire elements. The ratio of carbon dioxide emissions to economic or energy-related variables is used to determine the majority of an element’s carbon emissions efficiency. Comprehensive consideration is given to multi-factor carbon emission efficiency in the course of economic activity, which is frequently employed. Methods for measuring multi-factor carbon emission efficiency include ideal point cross efficiency, data envelopment analysis, and stochastic frontier analysis [12,13,14]. Economic growth, technical advancement, and urbanization are the key determinants of carbon emission efficiency. The increase in economic growth and technical innovation will both contribute to the enhancement of carbon emission efficiency [15,16]. The effect of urbanization on population and urban form on carbon emission efficiency is unknown [14]. Moreover, environmental regulations and energy architectures are significant determinants of carbon emission efficiency [17,18]. In terms of the characteristics of the temporal and spatial evolution of carbon emission efficiency, the carbon emission efficiency of the construction industry exhibits a high distribution trend in the east and a low distribution trend in the west [19], and the carbon emission efficiency of agriculture exhibits an overall upward trend, but the agricultural carbon emission efficiency varies greatly between regions, and the transportation industry has a higher carbon emission efficiency [20,21]. Although research on carbon emission efficiency has reached a quite advanced stage, social network analyses of carbon emission efficiency remain uncommon.

A carbon emission network is a social network analysis (SNA) technique that examines the complicated network interaction between cities’ carbon emissions. Existing research has identified substantial geographical connections between Chinese provinces in carbon emissions, with Tianjin, Zhejiang, Guangdong, Jiangsu, and Shanghai ranking among the most central locations [22]. Hebei and Inner Mongolia are the largest suppliers of carbon emissions, whereas Guangdong and Zhejiang are the largest consumers [23]. A great number of empirical and case studies have been undertaken on region-wide network drivers, particularly utilizing the quadratic allocation process (QAP) model and the exponential random graph (ERGM) model, in the context of expanding research on complex networks [24]. For instance, Bai et al. investigated the network structure and driving variables of carbon emissions in China and discovered that Henan is a region in need of critical governance [25]. The structure of carbon emission spatial association networks in the power sector of each Chinese province from 2005 to 2016 was then built by He et al. [5]. The carbon emission network disregards the factor movements between cities, and to a certain extent, because of the presence of invisible carbon, it also conceals the network features generated by factor flows [26,27]. Nevertheless, carbon emission efficiency occurs from the interplay of various components; hence, the network for carbon emission efficiency is more multidimensional. There is a clear contrast between the carbon emission network and the carbon emission efficiency network.

In summary, existing practices on carbon efficiency and networks are extensive. Yet importantly, there is potential for development in the analysis of carbon emission efficiency networks. First and foremost, existing research on carbon emission efficiency networks focuses mostly on the geographical interaction between a specific sector and the Yangtze River Economic Zone. In contrast to the Yangtze River Economic Belt, the Yellow River Basin is not an axial growth model based on geographical patterns and historical rules. The Yellow River Basin has a massive economic development gap, significant ecological and water resource issues, and more severe imbalanced and inadequate development tensions than the Yangtze River Economic Belt [28]. Therefore, a study of the Yellow River Basin’s carbon emission efficiency network is required. Second, the majority of network study viewpoints on carbon emission efficiency are provincial or municipal in nature. City clusters, however, as the highest spatial organization of cities in the process of industrialization and urbanization to higher levels of development in a particular region, are responsible for the aggregation and diffusion of various production factors and are the primary growth poles for regional economic development [29,30]. Consequently, it is vital to examine the network of carbon emission efficiency from the standpoint of City clusters. This research is not based on the study of a single city cluster, but rather on a comparative cross-sectional analysis of city clusters to examine the network structure for carbon emission efficiency. In light of this, the spatial relationship and spatial structure of carbon emission efficiency within the Yellow River Basin urban agglomeration are studied from a network perspective in order to improve the efficiency of carbon emissions and contribute to China’s realization of carbon peaks and carbon neutrality.

## 2. Materials and Methods

### 2.1. Study Area

**Figure 1:**
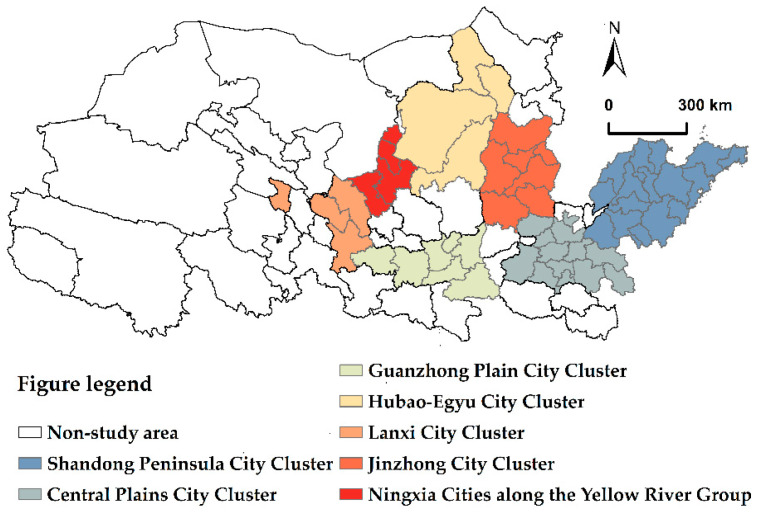
Study area.

The Yellow River begins in the Bayan Har Mountains in the Chinese province of Qinghai and flows through four geomorphic units. The river travels through nine provinces (regions), including Shanxi, Henan, and Shandong; the total area is 795,000 km^2^. The year-end resident population of the Yellow River Basin reached 421 million in 2020, with a total economic output of 25.39 trillion yuan. In August 2020, General Secretary Xi Jinping considered the Outline of the Plan for Ecological Protection and High-Quality Development of the Yellow River Basin (from now on referred to as “the Outline”) while presiding over a meeting of the Central Political Bureau. The Outline states, “The ecological protection and high-quality development of the Yellow River Basin should be taken as a thousand-year plan for the great rejuvenation of the Chinese nation.” Therefore, this article utilizes the seven city clusters of the Shandong peninsula city cluster, Central plains city cluster, Jinzhong city cluster, Guanzhong plain city cluster, Ningxia along the Yellow River group, Hubao-egyu city cluster, and Lanxi city cluster. Due to the city cluster’s little overlap in compositional shape and the difficulties of collecting data in certain county-level cities and ethnic minority autonomous prefectures, 55 cities were chosen as data collection samples, as seen in Figure 1. This article dates between 2006 and 2019. The information is derived from the “China Statistics Yearbook” and “China Energy Statistics Yearbook” as well as the statistics yearbooks of other cities. Due of the unavailability of individual cities and years, this study’s interpolation approach has been supplemented accordingly.

### 2.2. Measurement of Carbon Emission Efficiency

The traditional data envelopment analysis (DEA) model is based on a radial distance function that measures the target efficiency from a single input or output perspective. However, the radial condition is not fully satisfied in many cases when applied in practice. To address such non-expected outputs, the slack based model proposed by Tone is a non-radial, non-angle DEA model, which can solve the slack problem well by adding non-expected output variables and correcting for slack variables [31]. However, in the SBM model, it is difficult to compare the decision units when the efficiency value of multiple decision units is 1, thus causing bias in the final decision. The super slack-based model can decompose the decision units when the efficiency value is 1, thus comparing the effective decision units and enhancing the practical applicability of the model [32]. 

Assume a production system with n decision unit, each consisting of three input–output vectors: input, desired output, and undesired output, using m units of input to produce $S_{1}$ desired outputs and $S_{2}$ undesired outputs [33]. The three input–output vectors can be expressed as $x\in R^{m}$, $y^{g}\in R^{s_{1}}$, $y^{b}\in R^{s_{2}}$, where the matrices X, $Y^{g}$, $Y^{b}$ and are defined as follows:

$$X=\left[x_{1},x_{2},\cdots ,x_{n}\right]\in R^{m\times n},Y^{g}=\left[y_{1}^{g},y_{2}^{g},\cdots ,y_{n}^{g}\right]\in R^{S_{1}\times n},Y^{b}=\left[y_{1}^{b},y_{2}^{b},\cdots ,y_{n}^{b}\right]\in R^{S_{2}\times n}$$

Suppose X > 0, $Y^{g}$ > 0, $Y^{b}$ > 0. The set of production possibilities can be defined as $P=\left\{\left(x,y^{g},y^{b}\right)|x⩾X\theta ,y^{g}⩾Y^{g}\theta ,y^{b}⩽Y^{b}\theta ,\theta ⩾0\right\}$. θ denotes the weight vector.

The actual desired output is lower than the frontier ideal desired output level, and the actual non-desired output is higher than the frontier non-desired output level [34]. According to Tone’s SBM model, the SBM model that integrates the undesirable output into the evaluation decision unit $\left(x_{0},y_{0}^{g},y_{0}^{b}\right)$ based on the production possibility set is:

(1) $$\frac{1-\frac{1}{m}\sum_{i=1}^{m}\frac{S_{i}^{-}}{x_{i0}}}{1+\frac{1}{S_{1}+S_{2}}\left(\sum_{r=1}^{S_{1}}\frac{S_{r}^{g}}{y_{r0}^{g}}+\sum_{r=1}^{S_{2}}\frac{S_{r}^{b}}{y_{r0}^{b}}\right)},\;s.t.\;\{\begin{array}{l} x_{0}=X\theta +S^{-}\\ y_{0}^{g}=Y^{g}\theta -S^{g}\\ y_{0}^{b}=Y^{b}\theta -S^{b}\\ S^{-}⩾0,S^{g}⩾0,S^{b}⩾0,\theta ⩾0 \end{array},$$

where: $S=\left(S^{-},S^{g},S^{b}\right)$ indicates that input, expected output, and non -expected production relaxation amount. The target function value of the ρ is the efficiency value of the decision-making unit, which is between 0~1. For a given decision unit $\left(x_{0},y_{0}^{g},y_{0}^{b}\right)$, and only when ρ = 1, that is, $S^{-}=S^{g}=S^{b}=0$, the decision unit is effective. If 0⩽ρ<1 is inefficient, the evaluation unit is inefficient, and input and output need to be improved at this time. Since the above model is a non-linear model, it is not conducive to calculating the calculation of efficiency. Through the Charnes–Cooper transformation, the non-linear equation is converted into a linear model. The equivalent form is as follows:

(2) $$\rho =\min\tau =\min t-\frac{1}{m}\sum_{i=1}^{m}\frac{S_{i}^{-}}{x_{i0}},\;\;s.t.\;\{\begin{array}{l} 1=t+\frac{1}{S_{1}+S_{2}}\left(\sum_{r=1}^{S_{1}}\frac{S_{r}^{g}}{y_{r0}^{g}}+\sum_{r=1}^{S_{2}}\frac{S_{r}^{b}}{y_{r0}^{b}}\right)\\ x_{0}t=X\mu +S^{-}\\ y_{0}^{g}t=Y^{g}\mu -S^{g}\\ y_{0}^{b}t=Y^{b}\mu -S^{b}\\ S^{-}⩾0,S^{g}⩾0,S^{b}⩾0,\mu ⩾0,t>0 \end{array},$$

In the majority of studies evaluating efficiency, several choice units have their own “efficiency states”; thus, it is essential to distinguish these decision units and their influencing elements in efficiency ranking. To guarantee that efficiency analysis produces more fair efficiency evaluation values (SBM) and to incorporate the findings of Tone’s study in the literature, this thesis chooses the super slack-based model as the measurement object, which is stated as follows:

(3) $$\rho^{*}=\min\frac{\frac{1}{m}\sum_{i=1}^{m}\frac{{\bar{x}}_{i}}{x_{i0}}}{\frac{1}{S_{1}+S_{2}}\left(\sum_{r=1}^{s_{1}}\frac{{\bar{y}}_{r}^{g}}{y_{r0}^{g}}+\sum_{r=1}^{S_{2}}\frac{{\bar{y}}_{r}^{b}}{y_{r0}^{b}}\right)},\;\;s.t.\;\{\begin{array}{l} \bar{x}⩾\sum_{j=1,\neq k}^{n}\theta_{j}x_{j}\\ {\bar{y}}^{g}⩽\sum_{j=1,\neq k}^{n}\theta_{j}y_{j}^{g}\\ {\bar{y}}^{b}⩾\sum_{j=1,\neq k}^{n}\theta_{j}y_{j}^{b}\\ \bar{x}⩾x_{0},{\bar{y}}^{g}⩽y_{0}^{g},{\bar{y}}^{b}⩾y_{0}^{b},{\bar{y}}^{g}⩾0,\theta ⩾0 \end{array},$$

where: $\rho^{*}$ objective function takes the value of the decision unit efficiency. The range of values can be greater than 1. Other variables are defined in Equation (3), and the above models are built under the assumption of constant scale. 

We refer to the relevant literature and combine it with the actual situation in the Yellow River Basin. The capital and labor factors characterize the input and energy factors. The non-desired output indicator is characterized by carbon dioxide emissions, and the desired output is characterized by the gross domestic product (GDP). Table 1 defines the carbon emission efficiency network’s main indicators and calculation methods.

### 2.3. Exploratory Spatial Data Analysis Method

The ESDA largely uses spatial heterogeneity and spatial dependence analysis as the main tools. Spatial dependence analysis can be divided into two categories: one is Global Moran’s I, and the other is Local Moran’s I [37].

The global spatial autocorrelation is calculated as

(4) $${\mathrm{GlobalMoran}}^{\prime }s\;I=\frac{n\sum_{i=1}^{n}\sum_{j=1}^{n}W_{\mathrm{ij}}\left(x_{i}-\bar{x}\right)\left(x_{j}-\bar{x}\right)}{\left(\sum_{i=1}^{n}\sum_{j=1}^{n}W_{\mathrm{ij}}\right)\sum_{i=1}^{n}{\left(x_{i}-\bar{x}\right)}^{2}},\;(i\neq j),$$

The local spatial autocorrelation is calculated as

(5) $$LocalMoran^{\prime }s\;I=\frac{\left(x_{i}-\bar{x}\right)}{S^{2}}\sum_{j=1}^{n}W_{ij}\left(x_{j}-\bar{x}\right),(i\neq j),$$

where $X_{i}$, $X_{j}$ denotes the carbon emission efficiency of city i and j, respectively, and i≠j; $W_{ij}$ denotes the spatial location weight; x denotes the mean value of carbon emission efficiency; $S^{2}$ denotes the variance of carbon emission efficiency; n is the number of spatial units, which is 55 in this paper. Moran’s I takes values from −1 to 1, and the larger the absolute value of Moran’s I is, the stronger the spatial autocorrelation. If Moran’s I > 0, the spatial autocorrelation of urban carbon emission efficiency is positive. If Moran’s I < 0, the spatial correlation of urban carbon emission efficiency is negative.

### 2.4. Construction of the Carbon Emission Efficiency Network

#### 2.4.1. Determination of Spatial Correlations of Regional Carbon Emission Efficiency

In order to understand whether the carbon emission efficiency of the seven city clusters in the Yellow River Basin is characterized by a spatial network, a gravitational matrix needs to first be constructed [4]. A modified gravity model is introduced to measure the gravitational strength of the spatial association of carbon emission efficiency of the seven urban agglomerations in the Yellow River Basin. It is calculated as:

(6) $$Q_{ij}=\frac{eff_{i}\times eff_{j}}{D_{ij}^{2}}\times \frac{E_{i}}{E_{i}+E_{j}},$$

where: $Q_{ij}$ represents the gravitational force of carbon emission efficiency between cities i and j; $eff_{i}$, $eff_{j}$, $E_{i}$, and $E_{j}$ represents the carbon emission efficiency and gross domestic product of cities i and j; and $D_{ij}$ represents the geographical distance between cities i and j. 

#### 2.4.2. Exploring the Characteristics of Carbon Emission Efficiency Linkage Network Structure from the Perspective of Urban Agglomerations

In this paper, we measure the linkage of carbon emission efficiency between cities based on the gravitational model, record the gravitational values between cities, and then calculate the average value of gravitational values within each city cluster. If the actual value is larger than the average value, it will be 1; if not, it will be 0. Thus, the association matrix is obtained. Based on the construction of the correlation matrix, network density and central network potential are selected to analyze the structure of the carbon emission efficiency correlation network from the perspective of the city cluster as a whole, and centrality and cohesive subgroups are selected to analyze the characteristics of the carbon emission efficiency correlation network from the perspective of individual cities within the city cluster [38].

(1) Overall tightness of carbon emission efficiency network. The carbon emission efficiency correlation matrix calculated by the gravity model is a directed matrix, so the directed network density is to be explored and analyzed, and the calculation formula is:
  (7) E=L /[n(n−1)],
  where: E is the density of the carbon efficiency network, L is the number of actual links between cities, and n is the number of node cities of the network species.
(2) The overall centrality potential of the carbon emission efficiency network. In this paper, the degree centrality index reflecting the central potential is chosen to measure the degree of carbon emission network centrality and the expression of point-in (point-out) central potential.
  (8) $$C=\sum_{i=1}^{n}\left(c_{\max}-c_{i}\right)/\max\left[\sum_{i=1}^{n}\left(c_{\max}-c_{i}\right)\right],$$
  where: C is the degree of point-in (point-out) centrality of the carbon efficiency network; $c_{\max}$ is the maximum of the point-in (point-out) centrality of each city in the green network; $c_{i}$ is the point-in (point-out) centrality of the city i. The denominator reaches the maximum value $\left(n^{2}-3n+2\right)$ only when the network is a star-shaped network with n points.
(3) It uses the total number of connected cities with the remaining cities after binarization to measure the resource control ability of the individual’s centrality metric. The centrality of a node city in the network is equal to the number of the remaining nodes connected to the node. This paper uses the degree of point-out (the degree to which the carbon emission efficiency of this city affects other cities) and the degree of point-in (the degree to which this city is affected by the carbon emission efficiency of other cities) to represent the “out” and “in” of the carbon emission efficiency relationship, respectively. The greater the node’s centrality, the more central it is in the overall network.
(4) A cohesive subgroup is a method to divide the network nodes into small groups to study the relationship between subgroups. In this paper, the density matrix of each subgroup can be obtained by partitioning by the Concor method and then measuring the density of each subgroup separately. The three major urban clusters represent three blocks, respectively: compare the values in the density matrix of each subgroup in each block; the value more significant than the overall network density of the urban cluster is taken as 1, and the opposite scenario is 0, and finally, obtain the image matrix.

## 3. Results

### 3.1. Spatial and Temporal Spatial Variations of Carbon Emission Efficiency across City Clusters in the Yellow River Basin

#### 3.1.1. Timing Characteristics of Efficient Carbon Emissions within Context of City Clusters

**Figure 2:**
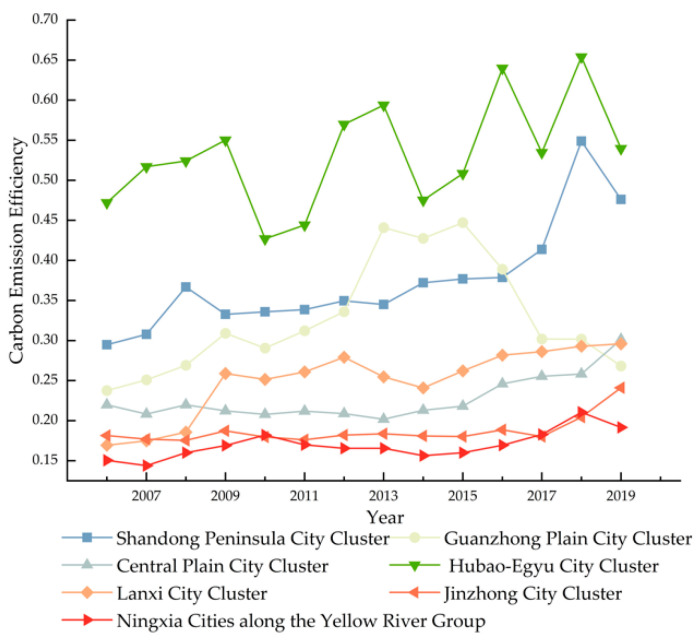
Changes in carbon emission efficiency by city cluster.

Figure 2 depicts the variations in the carbon emission efficiency of each city cluster from 2006 to 2019. From 2006 to 2019, the carbon emission efficiency of each city cluster fluctuates upward, with the greatest value increasing from 0.8665 in 2006 to 1.0297 in 2019, and the average growth rate reaching 41.08%. The shift in data suggests that the Yellow River Basin’s carbon emission management and optimization of production factors are successful. Nonetheless, there are two more considerations. First, as the difference in carbon emission efficiency across city clusters shrank from 2006 to 2019, the gradient differentiation features of each city cluster formed progressively. The Hubao-egyu city cluster was the first to graduate in 2007, and its carbon emission efficiency was considerably superior to that of the other city clusters. The second gradient consists of the Shandong peninsula city cluster and the Guan zhong plain city cluster, and the third gradient consists of the other city clusters. In 2019, the distance between the Hubao-egyu city cluster and the Shandong peninsula city cluster will have narrowed greatly, and the Guan zhong plain city cluster will have dropped to the third tier. However, it is difficult for city clusters with poor carbon emission efficiency to leave their gradient categories, which is not helpful to regional gap reduction. Second, carbon emission inefficient zones are progressively expanding, and the lowest growth rate compared to the average growth rate is just 0.0573%. Combined with Figure 2, it is discovered that city clusters with poor carbon emission efficiency account for a greater part of the sample, establishing a bottleneck for the Yellow River Basin’s carbon emission efficiency improvement. 

**Figure 3:**
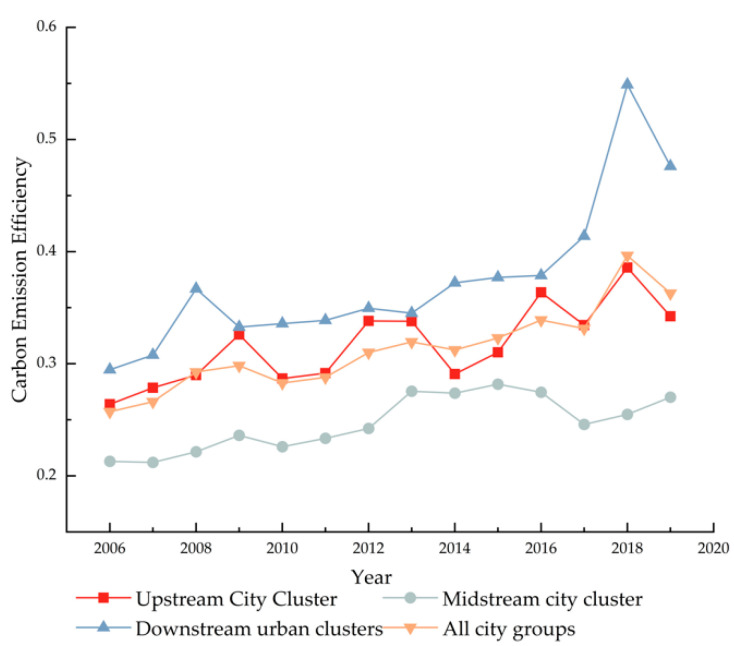
Changes in carbon emission efficiency of upstream and downstream urban agglomerations.

The seven city clusters are separated into up, middle, and downstream city clusters due to the contrasting geographic circumstances of the Yellow River’s upstream and downstream. The upstream city cluster includes Lanxi city cluster, Ningxia along the Yellow River group, and the Hubao-egyu city cluster; the midstream city cluster includes the Guanzhong plain city cluster, the Jinzhon city cluster, and the Central plains city cluster; and the downstream city cluster includes the Shandong peninsula city cluster. Figure 3 demonstrates that, from 2006 to 2019, the midstream city cluster in the Yellow River Basin has a poorer carbon emission efficiency than their upstream and downstream counterparts. Among these, the downstream city cluster, specifically the Shandong Peninsula city cluster, is the leader in terms of its high carbon emission efficiency. High carbon emission efficiency is directly associated with optimizing the industrial structure and enhancing economic growth in the downstream area. In the Yellow River Basin, the midstream city cluster is a region where heavy industrial bases are concentrated. Therefore, the city cluster in the midstream has features of high energy consumption and energy reliance. Simultaneously, industrial upgrading is complicated and urban transformation is challenging, reducing the efficiency of midstream carbon emission. In addition, the Chinese government launched the concept of high-quality development in 2017, which demands optimizing the energy structure, focusing on innovative development, and reforming the old approach to development. In 2018, however, all city groups improved their carbon emission efficiency due to a policy lag.

#### 3.1.2. City Clusters: Relevant Spatial Aspects of Carbon Emission Efficiency

**Figure 4:**
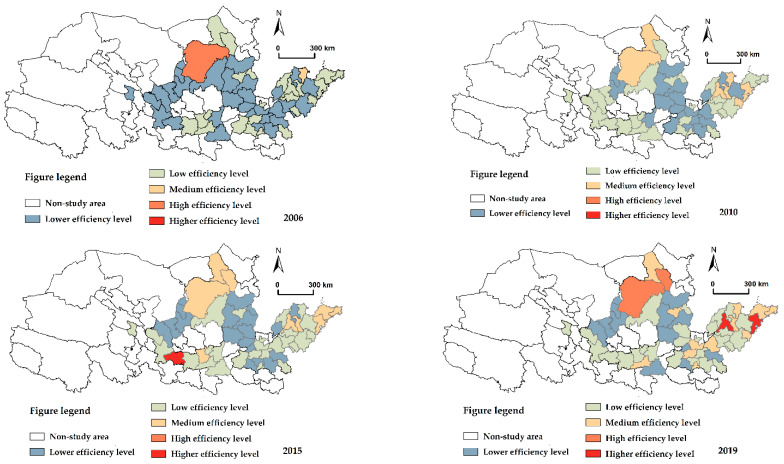
Spatial distribution of carbon emission efficiency in the Yellow River Basin.

Figure 4 illustrates the regional structure of carbon emission efficiency in the Yellow River Basin in 2006, 2010, 2015, and 2019. In order to facilitate a comparison of spatial dynamic development, this article is separated into five levels: lower efficiency level (0.01–0.22), low-efficiency level (0.22–0.44), medium-efficiency level (0.44–0.66), high-efficiency level (0.66–0.88), and higher efficiency level (0.88–1.10). Overall, the spatial distribution of carbon emission efficiency is very unbalanced, with a general downward tendency from east to west. The spatial divergence of carbon emission efficiency is mainly influenced by the level of economic development. In 2006, the geographical distribution of carbon emission efficiency in the Yellow River Basin was dominated by the lower efficiency and low-efficiency levels, with Ordos being the region with the greatest carbon emissions. In 2010, the distribution area of the lower efficiency level declined, whereas the distribution areas of the low-efficiency level and medium-efficiency level grew, with the highest concentration of medium-efficiency level in the cities of the Shandong Peninsula. In 2010, Tianshui saw the debut of its first high-efficiency level zones. Until 2019, the low-value region of carbon emission efficiency in the Yellow River Basin was mostly concentrated in Ningxia along the Yellow River Group and the Jinzhong city cluster, and it extends in all directions along the Yellow River’s main stem. Despite being smaller and more scattered, the city cluster of the Shandong Peninsula dominates the high-value area. The regional evolution of carbon emission efficiency is increasingly recalcitrant, and the pace of spatial change in localized regions is steadily diminishing. Throughout the study period, the spatial evolution patterns of the Shandong Peninsula city cluster, the Central Plains city cluster, and the Lanxi city cluster were superior to those of other city clusters, resulting in the evolution of carbon emission efficiency in the Yellow River Basin caused by the rigidity of spatial evolution. One of the main reasons for the inflexibility of the spatial evolution is the slower rate of efficiency improvement in the carbon-inefficient areas of the Basin compared to the high carbon-efficient areas.

**Figure 5:**
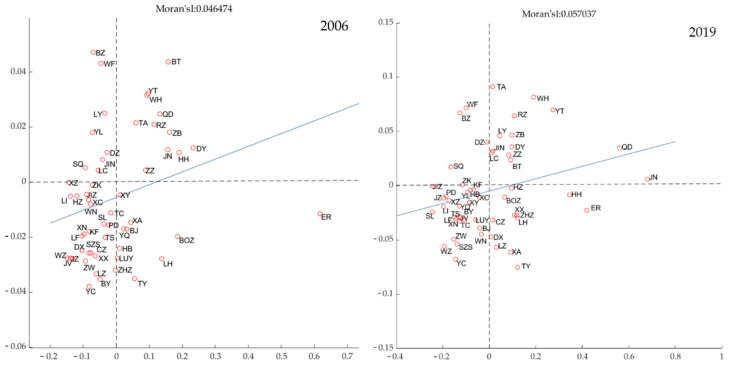
Local agglomeration characteristics of carbon emission efficiency in the Yellow River Basin.

As seen in Figure 5, certain cities exhibit local spatial agglomeration throughout time. Quadrant 1 is the high–high (H-H) agglomeration area, i.e., it is located inside the higher efficiency level and higher efficiency area, together with its neighboring regions. Quadrant 2 is the low–high (L-H) agglomeration region, meaning that the surrounding areas are more carbon-efficient the region itself, demonstrating a spatial catch-up effect. Quadrant 3 is the low–low (L-L) agglomeration, i.e., a region where both the region itself and the surrounding region have a low degree of efficiency and have poor spatial efficiency. Quadrant 4 is the high–low (H-L) agglomeration zone, meaning that its carbon emission efficiency is greater than that of the neighboring regions and geographically exhibits spillover effects. Comparing the distribution pattern of local geographical clustering of carbon emission efficiency in 2006 with 2019 reveals that the spatial characteristics of carbon emission efficiency in the Yellow River Basin emerge in two ways. First, the low–low agglomerations of carbon efficiency in China are now more interconnected and have grown slightly in size. Concurrently, the high–high agglomerations are consolidating further and beginning to grow in size. In addition, the investigation of the geographic distribution of agglomerations demonstrates a strong correlation between a region’s socioeconomic development level and its carbon emission efficiency [39]. In particular, a low–low agglomeration of Lanxi city cluster-Ningxia along the Yellow River Group was developed in the west in 2006. In the east, there is also a low–low agglomeration comprising Jinzhong city cluster, Zhongyuan city cluster, and Guanzhong city cluster. Dongying-Zibo-Jinan-Tai’an, Yantai-Weihao-Qingdao-Rizhao, and Zaozhuang, three high aggregation areas, established a city cluster on the Shandong peninsula. In addition, there is the high concentration area of Baotou city and Hohhot city. By the year 2020, the two preceding low–low agglomerations will have merged into one. Heze, Tongchuan, Dingxi, Changzhi, Xinxiang, and Lanzhou left the low–low agglomeration, bringing its size from 25 to 26 cities. In the meantime, Yulin, Hebi, Xuchang, Baoji, Xianyang, Luoyang, Yangquan, and Tongchuan enter the low–low metropolitan area. On the Shandong peninsula, the magnitude of the high–high agglomeration has increased, evolving from three scattered high–high agglomeration regions to an accumulation of high–high agglomeration areas. Linyi, Jining, and Liaocheng are the new cities in the high–high agglomeration areas, respectively. The Baotou-Hohhot high–high aggregation zone, however, mutated into the Baotou high–high aggregation zone. The second aspect is that the local Moran index rises from 0.0465 to 0.0570, indicating that carbon emission efficiency is more similar in local space, and the benefits of high–high agglomeration areas and low–low agglomeration areas continue to grow. The grab and overflow effects of low–high agglomeration areas and high–low agglomeration are, however, minimal. This proves the presence of the Matthew impact on the Yellow River Basin’s carbon emission efficiency. Due to this disparity in economic level and industrial structure, it is difficult to acquire endogenous power for the development of low-carbon technologies in the low-efficiency zone. The high-efficiency zone is limited by the industry’s influence on the expansion of development space. Therefore, it is difficult for the low-efficiency zone to obtain internal and external motivational support, which indicates that the low-efficiency zone lacks technical support for its carbon emission efficiency. 

### 3.2. Network Evolution Features of the Yellow River Basin City Clusters’ Carbon Emission Efficiency Network

#### 3.2.1. Preliminary Carving of Regional Carbon Emission Efficiency Correlation Net

**Figure 6:**
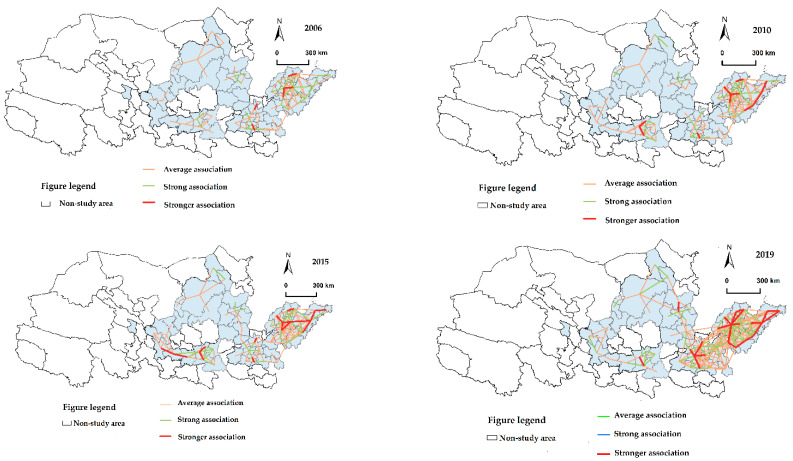
Spatial distribution pattern of carbon emission efficiency in the Yellow River Basin.

In this study, we use a modified gravity model to measure the association strength of carbon emission efficiency and ArcGIS software to classify the association strength as a weak association, average association, strong association, or stronger association, with the weak association’s gravitational line set to be invisible. As shown in Figure 6, from a temporal standpoint, the carbon emission efficiency of 55 cities in the Yellow River Basin varies substantially across years. The carbon emission efficiency network evolves from simple to complicated, and the relationship between the top reaches of the Yellow River Basin and the middle reaches of the Yellow River Basin tightens progressively. Simultaneously, the depth of the carbon emission efficiency network expands to the west, indicating a tendency of both internal inclusion and external growth. From a geographical viewpoint, the overall network structure of carbon emission efficiency in the Yellow River Basin is unevenly distributed, exhibiting strong hierarchical and regional features and a diminishing intensity of association and gravitational line density from east to west. In particular, in 2006, the network structure was predominantly weakly correlated, with a single correlation network structure primarily encompassing the Shandong peninsula city cluster and the Central plains city cluster; in 2010, the Guanzhong plain city cluster was extended to the west based on the axis, radiating to the Lanxi city cluster. Simultaneously, the relationship between small and medium-sized cities within the Yellow River Basin has been strengthened. In 2015, there was a rise in ties between the Central Plains city cluster and the Jinzhong city cluster. The overall carbon emission efficiency network had a substantial rise in density in 2019, with the weak relationship progressively transforming into a broad one. The downstream region’s network structure is taking form, including all the nearby small- and medium-sized cities, and the region’s carbon emission efficiency exhibits a degree of aggregation. With Zhengzhou, Taiyuan, and Xi’an serving as multi-cores, the midstream area creates a radial carbon emission efficiency network. The carbon emission efficiency links in the upstream area are becoming tighter, establishing an early “hourglass” network structure.

#### 3.2.2. Characteristics of Carbon Emission Efficiency Linkage Network Structure in the Perspective of City Clusters

From the viewpoint of city clusters, research on carbon emission efficiency contributes to the enhancement of carbon emission efficiency in the Yellow River Basin and increases the application of research findings.

##### Characteristics of the Overall Emission Efficiency Correlation Network Structure of Urban Agglomerations

**Figure 7:**
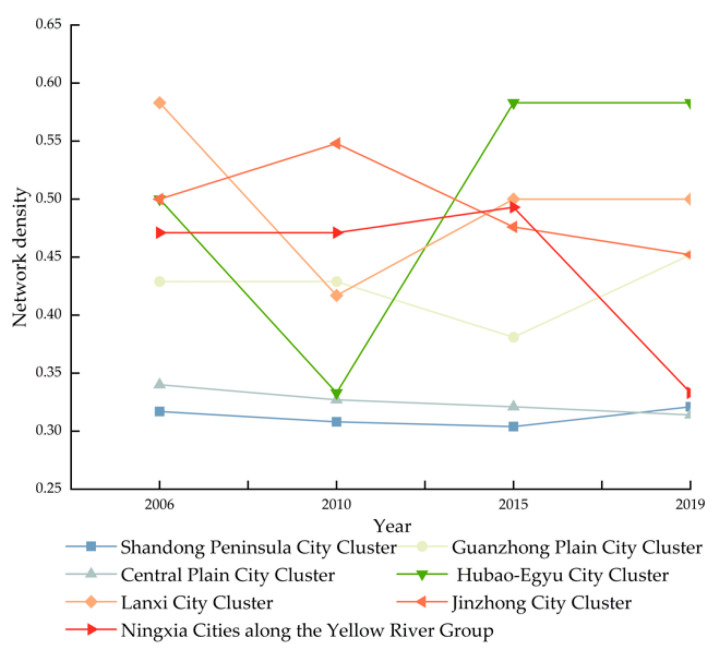
Net density of carbon efficiency in seven city clusters.

(1) For the examination of the overall stringency of carbon emission efficiency from 2006 to 2019, the number of linked nodes in the Shandong Peninsula city cluster is much higher than in other city clusters. In contrast to the highest figure, the real number of connections in the city cluster on the Shandong Peninsula city cluster is still modest. The maximum conceivable number of connections in the Shandong Peninsula city cluster is 240 (16 × 15); however, the greatest number of connections in the Shandong Peninsula city cluster during the research period was 77 (2019). Meanwhile, in each of the seven largest city clusters, some cities and the other cities have not formed any carbon emission efficiency association. In conclusion, the connections between carbon emission efficiency and the seven largest city clusters have much room for improvement. As shown in Figure 7, the network density of the Shandong Peninsula city cluster, the Guanzhong city cluster, and the Houbao city cluster increased between 2006 and 2019. The development trend of interregional synergy is favorable as the integration of urban clusters advances. The network densities of the remaining city clusters have all decreased to varying degrees, indicating that these four major city clusters do not pay sufficient attention to the improvement of carbon emission efficiency, and that factor flow between cities is insufficient, resulting in the stifling of the development of carbon emission efficiency improvement. The network density of all seven main city clusters is low, and the inter-city carbon emission efficiency connection is poor. The inter-city carbon emission efficiency linkage should be increased by modifying the network’s internal structure.
(2) Global examination of the centrality of carbon emission efficiency. As seen in Table 2, with the exception of Ningxia along the Yellow River Group, the point-in central potentials of the other six major city clusters from 2006 to 2019 are greater than the point-out central potentials, indicating that the seven major city clusters have weaker outward radiation ability and stronger inward cohesion ability. The point-in and point-out central potentials of the three major urban agglomerations of the Hubao city cluster, the Jinzhong city cluster, and Ningxia along the Yellow River Group in 2019 are lower than those in 2006, with the Hubao city cluster and Jinzhong city cluster showing an apparent decreasing trend, indicating that the carbon emission efficiency improvement of the region tends to diversify and the differences between cities are diminishing. In 2019, the point-in and point-out central potentials of the Central Plains city cluster are greater than they were in 2006, suggesting that the cohesiveness and external radiation of this urban agglomeration have risen. Both the 2019 point-in central potentials for the Shandong Peninsula city cluster and the Guanzhong city cluster are less than those of 2006. Nonetheless, the point-out central potentials of 2019 are larger than those of 2006 to varying degrees, indicating that the cohesion of the two city clusters has deteriorated while the radiation power has grown progressively. The point-in central potential of the Lanxi city clusters in 2019 is greater than in 2006; however, the point-out central potential is lower in 2019 than in 2016. Thus, the radiating power of the carbon emission efficiency of the Lanxi city clusters is diminished, and the attraction of resources is enhanced, showing that certain places within the urban agglomeration generate a “siphon effect” on the surrounding areas as a result of their dominant position.

##### Emission Efficiency Correlation Network Structure Characteristics within City Clusters 

Understanding the structural features of each node city in the network allows for the analysis of network issues and the promotion of the enhancement and optimization of carbon emission efficiency in the Yellow River Basin. The article picks typical city clusters from three important locations, including the upstream, midstream, and downstream Shandong Peninsula, Zhongyuan city cluster, and Lanxi city cluster, respectively. In addition, the network centrality and block models of the three largest metropolitan agglomerations are examined. Due to the limited number of cities in the Lanxi urban city cluster, it can only be evaluated for network centrality and not for cohesive subgroups.

**Figure 8:**
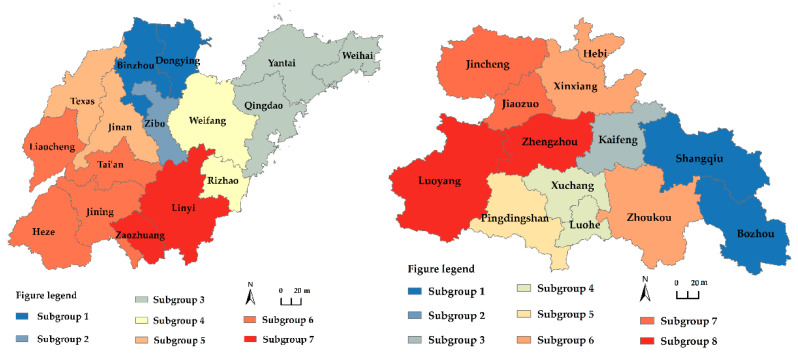
Division of two major urban agglomerations into cohesive subgroups.

**Figure 9:**
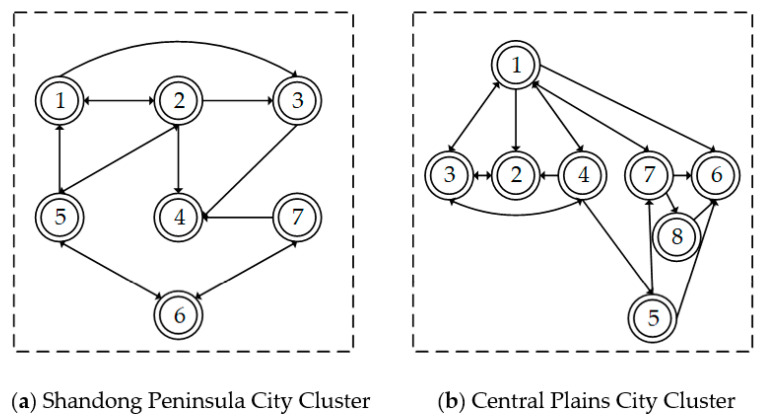
Simplification of carbon emission efficiency correlation networks in two major urban agglomerations.

(1) Analysis of cities with exceptional carbon emission efficiency. We concentrate on highlighting towns with outstanding carbon emission efficiency. The carbon emission efficiency network centrality of the Shandong Peninsula city cluster, Central Plains city cluster, and Lanxi city cluster was assessed using Ucinet program. Due to the uneven number of cities within the Yellow River Basin’s three main city clusters, only the top two cities are featured. As shown in Table 3, the top two cities of the three largest city clusters in terms of point-in and point-out degrees are identical. The top two cities are the carbon emission efficiency epicenters. As the inhaling area for carbon emission efficiency development, the top two cities also take the function of dispersal for this emission efficiency increase. The 2006–2019 Shan-dong Peninsula city cluster Taian takes the lead as the network’s center for carbon emission efficiency, as the development level of carbon emission efficiency is strong and has dominated the Shan-dong Peninsula city cluster. From 2006 to 2019, the top two cities in the Central Plains city cluster in terms of point-in and point-out rankings changed dramatically. Luohe ranked first in terms of point-in rating for four years, whereas Kaifeng ranked first in terms of point-out ranking for four years. Baiyin and Dingxi were the top two cities in terms of point-in rating for the Lanxi city cluster from 2006 to 2019. Therefore, this indicates that Baiyin and Dingxi carbon emission efficiency has a considerable pull on the surrounding regions. Lanzhou is one of the top two cities in the Lanxi city cluster ranking from 2006 to 2019. This demonstrates that Lanzhou city has a greater impact on the neighboring regions.
(2) Analysis of coherent subgroups of carbon emission efficiency. Ucinet software was used to conduct a block model analysis based on the correlation matrix of carbon emission efficiency for each city group in 2019. Figure 8 depicts the findings of the cohesive subgroups of the two main urban clusters, and Figure 9 shows the simplified “like matrix”. (1) Prominent subgroups: For instance, Zibo is the lone member of subgroup 2 of the Shandong Peninsula city cluster. Zibo is the industry leader in both input and output carbon emission efficiency and plays a crucial role. (2) The Bridge subgroup, such as Central plains city cluster 1 subgroup. As a representative, Shangqiu has a two-way communication connection with the main subgroup and is closely linked to Kaifeng and Jiaozuo. (3) For the net input subgroup: Pingdingshan, due to its proximity to the carbon emission efficiency subgroup, with Zhengzhou as the core. This subcluster’s resource components are more appealing to the subclusters that surround it. Their linkages are thus unidirectional, and the net output sub-group input impact is less than the output effect. In conclusion, the aforementioned subgroups have distinct tasks and labor divisions. Consequently, the network architectures of the two main urban clusters are notably different, although the characteristics of intra-city cluster links are identical to those of the overall linkages.

## 4. Conclusions, Recommendations, and Discussion

### 4.1. Conclusions

As an essential “energy basin” and rapid industrialization base in China, the Yellow River Basin’s green and low-carbon development is intimately tied to the achievement of China’s “double carbon” objective. This research evaluates the carbon emission efficiency of city clusters in the Yellow River Basin from 2006 to 2019 using the super SBM model and explores the regional and temporal development of carbon emission efficiency patterns. The modified gravity model is also utilized to analyze the network structure of carbon emission efficiency spatial correlation in the Yellow River Basin. It is observed that: (1) During the study period, the Yellow River Basin’s carbon emission efficiency increased considerably, but there is still potential for improvement, and there are considerable gaps across city clusters. In addition, the sluggish development rate in low-efficiency regions has created a bottleneck for the Yellow River Basin’s carbon emission efficiency improvement. Meanwhile, the geographical pattern of carbon emission efficiency demonstrates that the spatial evolution pattern of the Shandong Peninsula city cluster, the Central Plains city cluster, and the Lanxi city cluster is superior to that of other city clusters. (2) Throughout the term of study, the network correlation of the geographical correlation network of carbon emission efficiency in the Yellow River Basin rose, and the intensity of network connections shifted from weakly correlation-oriented to weakly correlation-oriented and averagely correlation-oriented. Regarding network density, The Shandong peninsula city cluster, Guanzhong city cluster, and Houbao urban city cluster had a growing network density trend, but the other city clusters exhibited the reverse tendency. In the meantime, the development pattern of carbon emission efficiency approaches equilibrium as time passes. (3) Analyzing the internal carbon emission efficiency of the Shandong peninsula city cluster, the Central Plains city cluster, and the Lanxi city cluster reveal over the research period that Tai’an, Luohe, Baiyin, and Dingxi have a greater impact on the surrounding regions. Although the association structures of the Shandong Peninsula city cluster and the Central Plains city cluster vary significantly, the association characteristics are the same.

### 4.2. Recommendations

(1) Taking into account the differences in carbon emission efficiency within the Basin, we select the most appropriate growth strategy based on local circumstances. The Basin should continue to strengthen the regional economic foundation, increase research and development of low-carbon technologies, enhance low-carbon innovation capacity, and promote the effective diffusion of low-carbon technologies based on the current development advantages and characteristics. For instance, using strategies such as “transport the natural gas from the West to the East“ and “to transmit the electricity from the western areas to East China,” the green and low-carbon energy transition may be successfully supported in the upper reaches of the Yellow River Basin. At the same time, provinces with a higher carbon efficiency in the Basin may also spread their energy-saving technologies and green innovation skills to other areas, fostering an increase in the Basin’s carbon emission efficiency.
(2) Concentrating on regions with poor carbon emission efficiency for the implementation of differentiated management methods, the region of low-value carbon emission efficiency must maximize the comparative benefits of resources and the environment. To avoid becoming a “refuge” for high-carbon businesses, we should also actively learn from advanced low-carbon technology and management expertise and accelerate the pace of industrial transformation and upgrading in the region. For instance, the industrial structure and reliance on coal and other resources of the Jinzhong city cluster are restrictions. With the advancement of low-carbon technology, we may therefore actively encourage the integration of scientific and technological elements into input variables and overcome the core contradiction of one coal and low-coal usage efficiency. By improving the structure of foreign trade, bringing sophisticated technology and management experience, and energetically promoting environmental sectors, the “Belt and Road” program can be used to increase the efficiency of carbon emissions.
(3) Maximizing the beneficial geographic spillover impact and cooperation to advance carbon reduction and efficiency in the basin, the economic development advantages of high carbon emission efficiency zones should be maintained while the innovation and development of low carbon technologies and industries are strengthened. High carbon emission efficiency zones should also give proactive technical and skilled assistance for the development of low-value zones, hence expediting the flow and distribution of high carbon economic development factors between areas. Cooperation among Yellow River Basin provinces (regions) should be enhanced. For instance, the Shandong peninsula and the Houbao-Egyu city cluster are encouraged to share their development experience of balancing economic output and carbon emissions. Additionally, cooperatively investigate low-carbon development pathways should be cooperatively investigated in order to establish diverse and clean energy structures.

### 4.3. Discussion

This study broadens the scope and methodology of previous research on carbon emission efficiency and offers some educational and practical insights into the solution for carbon emission efficiency that exists in the Yellow River basin. Nonetheless, this paper has certain drawbacks. First, the study examines, from the standpoint of urban clusters, the geographical distribution and correlation features of carbon emission efficiency in the Yellow River Basin. In the future, it will be necessary to delve deeper and compare data from various spatial scales. Second, this work does not examine the elements that influence carbon emission efficiency. Uncertainty exists about the elements that determine the network structure of carbon emission efficiency, which is also a future research path to be continually improved.

## Figures and Tables

**Table 1:** Index system for measuring carbon emission efficiency.

First-Level Indicators	Second-Level Indicators	Third-Level Indicators
Inputs	Labor	The labor factor is the total number of employed persons in the municipal area.
	Capital	The capital stock is expressed in terms of capital stock [35]. The capital stock is calculated using the perpetual inventory method with the formula: $K_{i,t}=K_{i,t-1}\left(1-\delta_{i,t}\right)+I_{i,t}$ where: $K_{i,t}$ denotes the capital stock of city i in year t (billion yuan); $\delta_{i,t}$ denotes the capital depreciation rate, and this paper follows the estimation of Zhang Jun et al. The depreciation rate is calculated using 9.6%: $I_{i,t}$ denotes the capital flow (billion yuan).
	Energy	The energy factor is natural gas, liquefied petroleum gas, social electricity, and total heat supply. The energy consumption is converted into standard coal due to the lack of uniform units. The conversion factors are 1.33 kg tec/m3, 1.7143 kg tec/kg, 0.1229 kg tec/(kW-h), and 0.03412 kg tec/(MIL- J) in order to referring to the General Rules for Calculation of Comprehensive Energy Consumption.
Expected outputs	GDP	The GDP was deflated using 2006 as the base period.
Unexpected outputs	Carbon dioxide emission	The total carbon emissions were calculated according to Wu et al. [36].

**Table 2:** Centrality of carbon efficiency in seven city clusters.

Indicators	City Cluster	2006	2010	2015	2019
Point to center potential (%)	Shandong Peninsula city cluster	30.22	31.11	38.67	29.78
	Central Plain city cluster	35.42	36.81	37.50	38.19
	Guanzhong Plain city cluster	66.67	66.67	52.78	63.89
	Hubao-egyu city cluster	66.67	5.710	55.56	55.56
	Lanxi city cluster	55.56	77.78	66.67	66.67
	Jinzhong city cluster	58.33	52.78	22.22	25.00
	Ningxia cities along the Yellow River Group	6.820	5.710	6.820	5.170
Point out the central potential (%)	Shandong Peninsula city cluster	8.89	16.89	10.22	15.56
	Central Plain city cluster	26.39	18.75	19.44	29.17
	Guanzhong Plain city cluster	8.330	8.330	33.33	25.00
	Hubao-egyu city cluster	22.22	5.710	11.11	11.11
	Lanxi city cluster	55.56	33.33	22.22	22.22
	Jinzhong city cluster	19.44	13.89	22.22	5.560
	Ningxia cities along the Yellow River Group	6.820	5.710	6.820	5.170

**Table 3:** Centrality of carbon emission efficiency networks in three major urban agglomerations.

Year	Sorting	Shandong Peninsula City Cluster				Central Plain City Cluster				Lanxi City Cluster			
		Point-In Degree	Score	Point Out the Degree of Deviation	Score	Point-In Degree	Score	Point Out the Degree of Deviation	Score	Point-In Degree	Score	Point Out the Degree of Deviation	Score
2006	1	Taian	9.0	Taian	6.0	Luohe	8.0	Kaifeng	7.0	Baiyin	3.0	Xining	3.0
	2	Zibo	8.0	Zibo	6.0	Kaifeng	7.0	Zhengzhou	6.0	Dingxi	3.0	Lanzhou	2.0
2010	1	Taian	9.0	Taian	7.0	Xuchang	8.0	Kaifeng	6.0	Dingxi	3.0	Lanzhou	2.0
	2	Zibo	8.0	Zibo	6.0	Luohe	8.0	Zhengzhou	6.0	Baiyin	2.0	Dingxi	1.0
2015	1	Taian	10.0	Rizhao	6.0	Kaifeng	8.0	Luoyang	6.0	Dingxi	3.0	Lanzhou	2.0
	2	Zibo	8.0	Zibo	6.0	Luohe	8.0	Kaifeng	5.0	Baiyin	2.0	Xining	2.0
2019	1	Taian	9.0	Taian	7.0	Luohe	8.0	Luoyang	7.0	Dingxi	3.0	Lanzhou	2.0
	2	Zibo	8.0	Zibo	7.0	Xinxiang	7.0	Kaifeng	5.0	Baiyin	2.0	Xining	2.0

## Data Availability

Data available on request due to restrictions privacy. The data presented in this study are available on request from the corresponding author.

## Abbreviation

Full of City Name	Abbreviation of City Name	Full of City Name	Abbreviation of City Name	Full of City Name	Abbreviation of City Name
Qingdao	QD	Pingdingshan	PD	Erdos	ER
Jinan	JN	Xinxiang	XX	Baotou	BT
Zibo	ZB	Jiaozuo	JZ	Yulin	YL
Zaozhuang	ZZ	hebei	HB	Lanzhou	LZ
Yantai	YT	Xuchang	XC	Xining	XN
Weifang	WF	Luohe	LH	Dingxi	DX
Jining	JIN	Shangqiu	SQ	Baiyin	BY
Linyi	LY	Zhoukou	ZK	Taiyuan	TY
Taian	TA	Jincheng	JV	Yangquan	YQ
Liaocheng	LC	Bozhou	BOZ	Changzhi	CZ
Heze	HZ	Xi’an	XA	Xinzhou	XZ
Dezhou	DZ	Tongchuan	TC	Jinzhong	JIZ
Binzhou	BZ	Baoji	BJ	Lvliang	LI
Dongying	DY	Xianyang	XY	Linfen	LF
Weihai	WH	Weinan	WN	Yinchuan	YC
Rizhao	RZ	Shangluo	SL	Shizuishan	SZS
Zhengzhou	ZHZ	Tianshui	TS	Wuzhong	WZ
Kaifeng	KF	huhehaote	HH	Zhongwei	ZW
Luoyang	LUY				
